# Locally Oriented Scene Complexity Analysis Real-Time Ocean Ship Detection from Optical Remote Sensing Images

**DOI:** 10.3390/s18113799

**Published:** 2018-11-06

**Authors:** Yin Zhuang, Baogui Qi, He Chen, Fukun Bi, Lianlin Li, Yizhuang Xie

**Affiliations:** 1 School of Electronics Engineering and Computer Science, Peking University, Beijing 100087, China; zhuangyin640829@163.com (Y.Z.); lianlin.li@pku.edu.cn (L.L.); 2 Beijing Key Laboratory of Embedded Real-Time Information Processing Technology, Beijing Institute of Technology, Beijing 100081, China; qibaogui@bit.edu.cn (B.Q.); chenhe@bit.edu.cn (H.C.); 3 Department of Electronic and Information Engineering, North China University of Technology, Beijing 100144, China; bifukun@163.com

**Keywords:** feature clustering, optical remote sensing, ship detection, scene partition, saliency

## Abstract

Due to strong ocean waves, broken clouds, and extensive cloud cover interferences, ocean ship detection performs poorly when using optical remote sensing images. In addition, it is a challenge to detect small ships on medium resolution optical remote sensing that cover a large area. In this paper, in order to balance the requirements of real-time processing and high accuracy detection, we proposed a novel ship detection framework based on locally oriented scene complexity analysis. First, the proposed method can separate a full image into two types of local scenes (i.e., simple or complex local scenes). Next, simple local scenes would utilize the fast saliency model (FSM) to rapidly complete candidate extraction, and for complex local scenes, the ship feature clustering model (SFCM) will be applied to achieve refined detection against severe background interferences. The FSM considers a fusion enhancement image as an input of the pulse response analysis in the frequency domain to achieve rapid ship detection in simple local scenes. Next, the SFCM builds the descriptive model of the ship feature clustering algorithm to ensure the detection performance on complex local scenes. Extensive experiments on SPOT-5 and GF-2 ocean optical remote sensing images show that the proposed ship detection framework has better performance than the state-of-the-art methods, and it addresses the tricky problem of real-time ocean ship detection under strong waves, broken clouds, extensive cloud cover, and ship fleet interferences. Finally, the proposed ocean ship detection framework is demonstrated on an onboard processing hardware.

## 1. Introduction

Ocean ship detection is an active research field in remote sensing technology. This field is primarily applied to fishery management, vessel salvaging, and naval warfare applications. For several decades, synthetic aperture radar (SAR) images have typically been used for ship detection research because SAR images can be obtained at any time in any weather conditions [1,2,3,4]. However, compared to SAR images, optical remote sensing images can provide more detailed and visible characteristics for ship detection and classification [5,6]. Therefore, most researchers have recently focused on ship detection based on optical remote sensing images, in order to realize a quick, accurate, and automatic detection framework [7,8,9,10,11,12,13,14,15,16,17,18,19,20]. Simply stated, the typical optical ocean ship detection framework has two stages which are ship candidate extraction and confirmation. In the ship candidate extraction stage, the performance is affected by the gray level or shape diversification of ships and complex background interference. If there is a low contrast ratio, small targets or ship fleets may not be detected. Next, a complex background that includes extensive cloud cover, broken clouds, and strong waves would produce many false alarms in the ship candidate extraction stage. Following the candidate extraction stage, the candidate conformation needs a powerful and suitable feature description method to distinguish ships from suspected candidates using a binary classifier. However, there are no effective feature descriptions to better discriminate ships. Therefore, in the face of challenges of an effective feature description and complex background interference, and considering the timeliness requirement, a more reliable real-time practical ocean ship detection application is required.

The current existing ocean ship detection approaches are more effective in quiet sea conditions or simpler ocean scenes, and they perform poorly when the scenes include complex background interferences. In addition, most existing methods also require complicated computations which make it difficult to satisfy the real-time processing requirement. For example, Zhu et al. [8] proposed a novel hierarchical ship detection method based on shape and texture features that combined some simple shape features with local multiple patterns (LMP) to achieve a hierarchical feature description, and then, a semi-supervised classification was used to eliminate false alarms. However, this method [8] would not work on complex ocean scenes (i.e., when facing extensive cloud cover, broken clouds, strong waves, or a fleet of ships) because of the limitations of the feature description capability. Muller R. et al. [17] proposed a near real-time optical ship detection framework which was composed of multiple hierarchical Haar-like features. Next, Adaboost and a cascade classification strategy were used to detect ships and eliminate false alarms, but the artificially designed hierarchical Haar-like features were also invalidated by the changing characteristics of ships and complex background interference. Recently, several researchers are focused on saliency analysis methods. Qi et al. [11] created an unsupervised ship detection model, which is based on the phase spectrum of a Fourier transform (PFT) cascading saliency analysis with a ship histogram orientation gradient (S-HOG) descriptor. Bi et al. [9] employed the pulse cosine transform (PCT) to achieve ship candidate saliency analysis, and the context model with a modified scale invariant feature transform (SIFT) is employed to eliminate false alarms and confirm a ship candidate. Fang Xu et al. [19] proposed a saliency model based on wavelet transform analysis, and improved entropy and pixel distribution discrimination features were employed to remove false alarms using a multilevel structure. Chao Dong et al. [18] proposed a ship detection framework was based on variance statistical feature saliency analysis, and a rotation-invariant global gradient description for the SVM classifier. These methods have a common property, which is that they use the ship’s saliency feature analysis to obtain the suspected candidates. However, the saliency analysis methods are often unable to identify ship candidates under complex background scenes. The saliency model is more suitable for simple ocean scenes. With respect to complex ocean scenes, the complicated saliency model could be partially effective, but it cannot meet the real-time requirement. To improve complex ocean scene ship detection performance, many researchers have focused on high level feature descriptions to avoid the drawbacks of artificially designed features. Tang et al. [13] proposed a framework in the compressed domain using the deep belief networks (DBN) and an extreme learning machine (ELM) to achieve optical ocean ship detection. While Zou et al. [15] built a singular value decomposition convolutional network (SVDnet). However, since the application is constrained when using low or medium resolution optical remote sensing images covering a large oceanic field, the ship targets would not be characterized by rich feature information. Thus, the small and relatively weak targets would limit the effectiveness of the feature description methods described previously [13,15], which can cause these methods to perform poorly. Furthermore, in addition to a low contrast ratio and small scale, complex background interference can also impact the performance of ocean ship detection. Conversely, previously described methods [13,15] need large ship samples to train the neural network, but a large amount of manually annotated remote sensing data is not usually available. In general, the state of the art methods for ocean ship detection using optical remote sensing images perform poorly when faced with complex background interferences. The main problems are the ship’s discriminative feature descriptions when analyzing low or medium resolution images and processing large view field scene, the timeliness when high precision ship detection performance is necessary.

**Figure 1:**
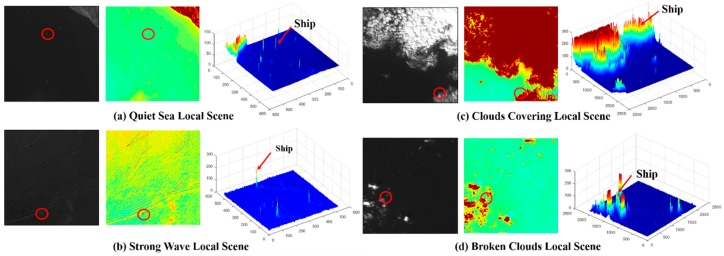
Examples of ocean ship detection in full scenes of ocean optical remote sensing images. (**a**) is a local scene with a quiet sea; (**b**) is a local scene with strong wave; (**c**) is a local scene with cloud cover; and (**d**) is a local scene with broken clouds.

In this paper, as previously mentioned, we focus on the problems of ocean ship detection and propose a novel ship detection framework that can simultaneously satisfy the detection accuracy and real-time processing requirements. In our opinion, full ocean optical remote sensing images can be classified into two types of simple and complex local scenes. In this instance, the simple local scenes occur in the case of quiet seas, while complex local scenes represent situations with a fleet of ships, strong waves, and/or clouds. Many detection methods fail when analyzing complex local scenes. Therefore, the proposed ship detection framework is focused on these complex local scenes to improve the overall detection performance. Then, most general full ocean remote sensing scenes contain many simple or complex local scenes as shown in Figure 1. Thus, we have designed a locally oriented scene complexity real-time analysis detection method to meet the practical ocean ship detection system requirements.

**Figure 2:**
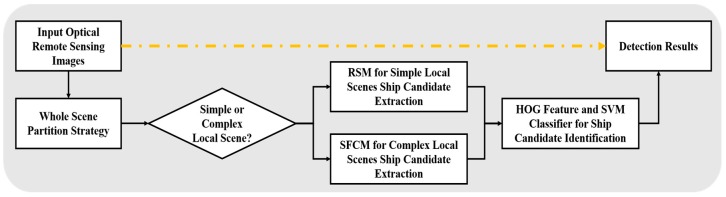
Workflow of the proposed ocean ship detection framework.

Figure 1 shows several general ocean optical remote sensing scenes, in which the strong waves, cloud cover, and broken clouds seriously influence the detection performance. Overcoming these complex interferences can improve the ocean ship detection performance. Therefore, we establish a rule for a full optical remote sensing scene partitioning strategy to separate a scene into two types of local scenes. Then, based on the local scene’s characteristics for the simple local scenes, a novel rapid saliency model (RSM) is employed to achieve ship candidate extraction and fast processing of the simplest local scenes. Next, for the complex local scenes, the ship feature clustering model (SFCM) is proposed to complete the accurate ship detection. Finally, the HOG feature descriptor trained SVM classifier is used for candidate identification. This proposed ship detection framework is shown in Figure 2.

In this paper, the contributions can be summarized in three points. First, this paper proposed a novel ocean ship detection workflow which is based on the locally oriented scene complexity analysis method to meet real-time processing requirements. Second, this paper improved an RSM to achieve rapid and accurate ship detection in simple local scenes. Third, this paper proposed a novel SFCM realizing ocean ship detection in complex local scenes. Therefore, the following sections present a detailed introduction to the locally oriented scene complexity analysis, and the RSM and SFCM ship detection models.

## 2. Optical Remote Sensing Ocean Ship Detection

### 2.1. Locally Oriented Scene Complexity Analysis

Since ocean ship detection is sensitive and has a timeliness requirement, real-time processing is very important in military and civil applications. In addition, full scene ocean optical remote sensing images are usually divided into many blocks to achieve ocean ship detection, which is a popular method in real-time ocean ship detection frameworks. However, the ocean optical remote sensing image sub-blocks contain many situations. For example, these blocks include quiet sea, strong waves, heavy cloud cover, broken clouds, and so on. The various detection methods vary in performance under local conditions resulting in failure in some complex local scene situations, leading to missing targets or false alarms. If we can find a way to distinguish these complex local scene blocks, we can apply appropriate treatment to ensure a high overall detection performance. Therefore, for full scene ocean optical remote sensing images, we aim to improve both the speed of simple local scene detection and the accuracy of complex local scene detection in order to satisfy the real-time ship detection system requirements.

In this paper, the fast scene partitioning strategy is proposed to achieve the sub-block’s local scenes classifications. Due to the illumination problem in optical remote sensing images, we have chosen the texture descriptor to analyze the local scenes’ characteristics. Then, the blocks are defined according to whether they are simple or complex local scenes. Related to the texture characteristic description, first we calculate the gradient feature map from the gray level local scenes, which can be expressed as Equations (1)–(3):

(1) $$G_{h}=\frac{1}{wh}\sum_{x=1}^{w}\sum_{y=1}^{h}\left[1-{\left(\frac{I_{x+1,y}-I_{x,y}}{BL}\right)}^{2}\right].$$

(2) $$G_{v}=\frac{1}{wh}\sum_{x=1}^{w}\sum_{y=1}^{h}\left[1-{\left(\frac{I_{x,y+1}-I_{x,y}}{BL}\right)}^{2}\right].$$

(3) $$G\left(i,j\right)=\sqrt{G_{h}^{2}+G_{v}^{2}}.$$

In this instance, in Equations (1) and (2), *w* and *h* are the length and width of local scenes, respectively. Here, *BL* is the input data quantization level. *G_h_* and *G_v_* are the horizontal and vertical gradients, respectively. *x* and *y* are the index coordinates in local scenes, and *I_x,y_* is the gray value of index coordinates (*x*,*y*). Then, the gradient feature map *G*(*i,j*) is calculated as in Equation (3). The texture feature would be expressed by summing the local gradient features, as shown in Equation (4).

(4) $$Ir=\left[\begin{array}{ccc} G(\frac{n+1}{2},\frac{n+1}{2}) & \ldots & G(\frac{n+1}{2},h-\frac{n+1}{2})\\ \vdots & \ddots & \vdots \\ G(w-\frac{n+1}{2},\frac{n+1}{2}) & \cdots & G(w-\frac{n+1}{2},h-\frac{n+1}{2}) \end{array}\right].$$

**Figure 3:**
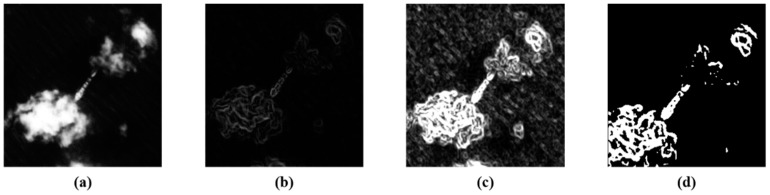
Texture feature map construction. (**a**) is the original local scene; (**b**) is the gradient feature map; (**c**) is the texture feature map; and (**d**) is a binary image.

**Figure 4:**
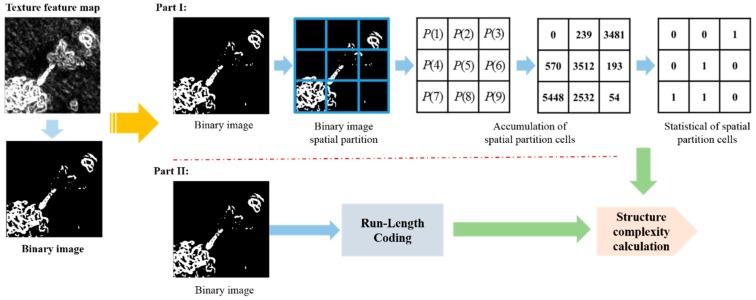
Local scene partition method for scene characteristic classification.

In Equation (4), *w* and *h* are the size of local scenes. *G* is the gradient feature map, which is generated from Equation (3). In this equation, *n* is the size of the sliding window, which defines the summation of the gradient feature values of the gradient feature map. *I_r_* is the constructed texture feature map that can be used to avoid the illumination problem which can enhance optical remote sensing images without low contrast ratio. If ships, broken clouds, islands, and strong waves appear in local scenes, these objects can be highlighted in the constructed texture feature map because they have rich texture features. Therefore, the proposed locally oriented scene analysis method utilizes the OTSU thresholding algorithm [21] to generate the binary image from the texture feature map to analyze local scenes’ characteristics. The texture feature map construction process [22,23,24] is shown in Figure 3, and the binary texture feature map analysis method is shown in Figure 4.

In Figure 3c, we can see that the texture feature map clearly shows the texture information of local scenes. Before applying the OTSU algorithm, the strong textures are set to “1”, and the weak textures to “0”. Here, Figure 3d is the binary image, prepared for the local scene characteristic analysis.

Figure 4 shows an example of local scene characteristics analysis. It uses a 3 × 3 partitioning strategy to analyze nine cells texture information in part I. Then, it sums *cell*(*N*) to evaluate the texture quality, here, *N* equal to 1 to 9. The evaluation processes can be expressed as Equations (5)–(7):

(5) $$cell(N)=\sum_{i=1}^{r}\sum_{j=1}^{c}I_{binary}(i,j).$$

(6) $$P(N)=\{\begin{array}{c} \begin{array}{ccc} 1, & if & cell(N)\geq \frac{size(cell)}{3}. \end{array}\\ \begin{array}{ccc} 0, & if & cell(N)<\frac{size(cell)}{3}. \end{array} \end{array}.$$

(7) $$D=\frac{\sum_{i=1}^{M}P(N)}{M}.$$

**Figure 5:**
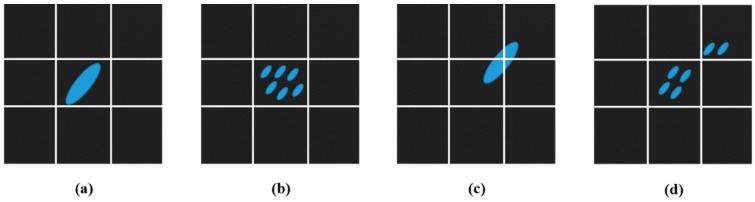
Structural and textural complexity analyses. (**a**) has a lower textural distribution ratio *D* and a simple structure; (**b**) has a low textural distribution ratio D and a complex structure; (**c**) has a more complex textural distribution ratio *D* and a simple structure; and (**d**) has a more complex textural distribution ratio *D* and a complex structure.

In Equation (5), *N* is the index of the cell number, and (*i*,*j*) is the coordinate in the binary image *I_binary_*. Then, Equation (5) can achieve the accumulation of each local scene’s spatial partition cells. The accumulation result Equation (6) is used to evaluate the significant textural information of the cells. When the accumulation is more than a third of one cell, this sub-cell is set to “1”, which is the textural statistical process, since there are “1 s” or “0 s” in the local scene textural distribution based on 3 × 3 partitioning. In Equation (7), *M* is the total number of local scene partition cells, and *D* is the distributional statistical value of the textural information appearing in each cell which can indicate whether a large variation of texture occurs in the current local scene. If this local scene has large scale texture, it would be classified as a complex local scene, and *D* would be endowed with a large number following the definitions of Equations (5)–(7). Here, the local scene structure also affects the complexity judgment. The examples of structural complexity analysis are shown in Figure 5.

From Figure 5a,b, we can see that these local scenes have the same *D* ratio, but (a,b) have significantly different structures. Then, the same phenomenon is seen in (c,d). Therefore, the object’s structure is complete or dispersed, which is also an important element in evaluating the complexity of local scenes. In this paper, Run-Length coding [24] is employed to evaluate the object’s structural complexity index *R* which is shown in Equation (8).

(8) $$R=D\times \left(\frac{L_{RL}}{A}+\frac{A}{W\times H}\right).$$

In Equation (8), *L_RL_* is the length of the Run-Length coding [24] which can investigate structure complexity of object’s binary images, and *A* is the area of the object’s texture in the binary image. *W* and *H* are the size of the local scene. *D* is the textural distribution ratio which was previously defined. We can use the index *R* in Equation (8) to rapidly separate a full ocean optical remote sensing scene into local scenes. The index *R* considers the textural spatial distribution and the structural complexity analysis of local scenes. To achieve fast scene partition, *R* would be a flexible threshold value in order to meet the real-time processing system requirement. If the local scene has a smaller *R*, it would be defined as a simple local scene, and if it has a larger *R*, it would be defined as a complex local scene. Here, simple local scenes contain fewer targets and/or a quiet sea, and complex local scenes include large cloud cover, many broken clouds, and/or a fleet of ships. Due to the real-time processing requirement, the rapid saliency model (RSM) is applied to simple local scenes, and the accurate ship feature clustering model (SFCM) is applied to complex local scenes. Next, through the RSM and SDCM, the proposed ship detection framework can achieve the rapid and accurate extraction of suspected ship candidates.

### 2.2. RSM for Simple Local Scene Ship Candidate Extraction

We focus on improving the simple local scene ship detection speed to meet the real-time processing system constraint. In simple local scenes, the ship target is obviously different from the background. However, most methods use a complex detection algorithm model, which leads to significant processing time which is not suitable for a real-time processing system [18,19,20]. In this section, a novel saliency model, RSM, is proposed. RSM comprehensively utilizes spatial and frequency domain information to generate the saliency map because of its good performance in simple local scenes [25,26,27,28]. The saliency map can be calculated using Equations (9)–(11).

(9) $$M_{I}=f_{b}\left(I\right)+f_{t}\left(I\right).$$

(10) $$P=sign\left(F\left(M_{I}\right)\right).$$

(11) $$SM=G*{\left(abs\left(F^{-1}\left(P\right)\right)\right)}^{2}.$$

**Figure 6:**
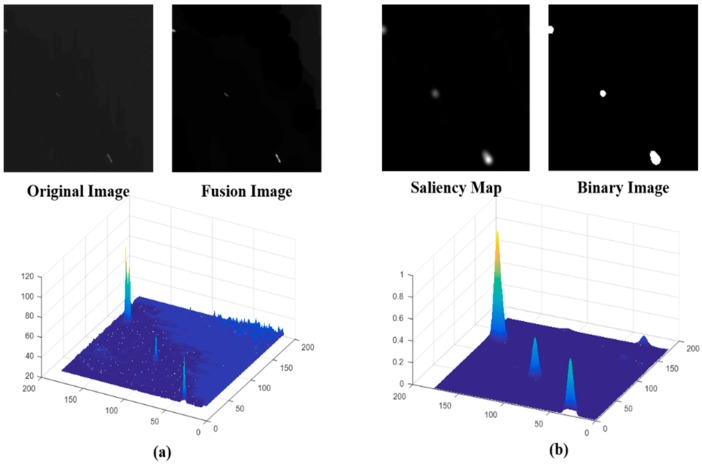
Rapid saliency model (RSM) for simple local scene ship detection. (**a**) is the original image and fusion image analysis and (**b**) is the saliency map and binary image analysis.

In (9), *I* is the defined simple local scene, and *f_b_*(·) and *f_t_*(·) are the bottom and top hat operations, respectively. The bottom and top hat operations can keep brightness and darkness objects in the transformation image and also enhance the difference between ships and background. These operations can prevent missing target ships caused by a weak gray level. *M_I_* is the fusion image that combines the bottom hat and top hat features. We employ the Fourier transform *F*(·) and *sign*(·) to obtain the responses in the frequency domain. Next, *abs*(·) and *F*^−1^(·) are used to get the pulse responses, and the square operation enhances the pulse responses. Finally, the two-dimensional Gaussian low pass filter *G* is employed to generate the saliency map, which can produce the ship candidates using the OTSU algorithm [23]. An example performance of an RSM is shown in Figure 6.

**Figure 7:**
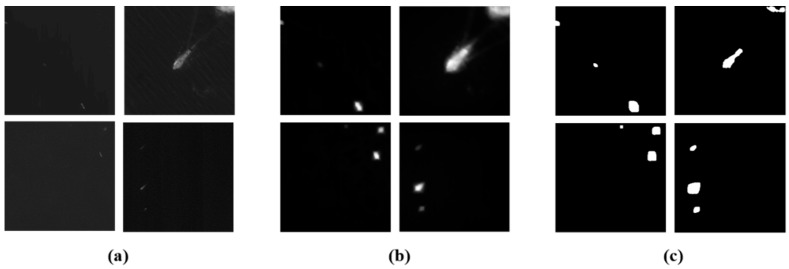
RSM ship detection performance. (**a**) Original simple local scenes; (**b**) RSM saliency map; and (**c**) ship candidate extraction results.

In Figure 6, we can see that our proposed RSM has better performance with low contrast ships in simple local scenes. Furthermore, it is also valid for one ship target that is divided into several non-overlapping local scenes. The RSM for simple local scene ship detection is shown in Figure 7. Where, we can see that the ship target and parts of ships are highlighted. The OTSU algorithm is applied to the saliency map for ship candidate extraction. All of the simple local scenes in Figure 7 show that the RSM has a good performance for any sized ships and parts of ships in different local scenes. Therefore, using our proposed RSM can rapidly and accurately extract ship candidates. The quantitative analysis of the RSM will be discussed using detailed examples and the discussion section.

### 2.3. SFCM for Complex Local Scene Ship Candidate Extraction

**Figure 8:**
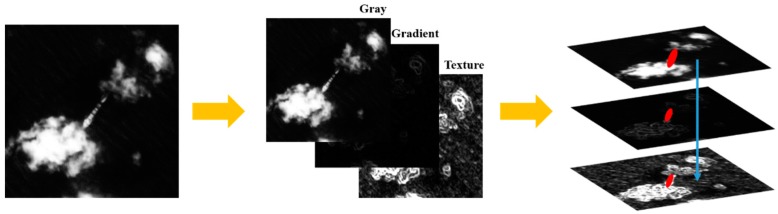
Procedure of generating a synthetic image.

For ship detection in complex local scenes, which is the most challenging task for ship detection, many methods perform poorly because of scene information interference. The major problem is focusing on the discriminative feature description of ocean ships [29,30,31,32,33,34,35,36]. For the SFCM, we build a synthetic information image that has three channels similar to RGB images to increase the descriptive dimension of each pixel feature vector. Then, each pixel of the synthetic information image includes gray, gradient, and textural feature information, which is shown in Figure 8. 

The gradient feature map that is generated by Equation (3) is added to the synthetic image, and the textural feature map that is produced from Equation (4) is also added to enhance the ship feature description. Then, the original one-dimensional feature is projected into the three-dimensional feature space. Which can better enable distinguishing the ship target from the background interferences. However, the high dimensional feature would increase the time taken for the ship feature clustering calculation. Therefore, in this paper, considering the real-time processing requirement, we use three-dimensional features to build the ship clustering model. Then, the proposed SFCM can be expressed as Equation (12).

(12) $$\sqrt{{\left(G-a\right)}^{2}+{\left(S-b\right)}^{2}+{\left(T-c\right)}^{2}}=d.$$

**Figure 9:**
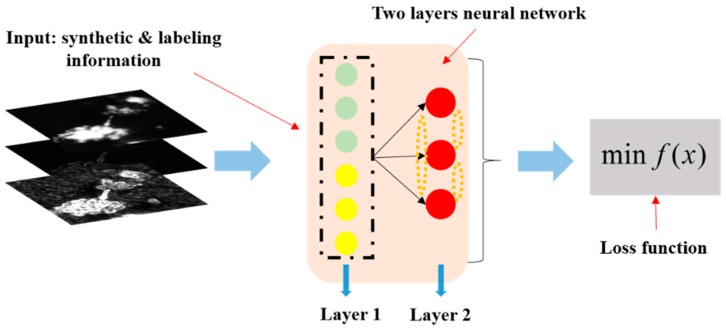
The generation process of the one-dimensional topological map.

In Equation (12), *a*, *b*, and *c* are the feature clustering center coordinates and *G*, *S*, and *T* are the values from the feature vector in the three-dimensional feature space. *d* is the controlling distance between the input feature vector and the clustering center coordinate. Here, to automatically get the clustering center coordinate (*a*, *b*, and *c*) and the controlling distance *d*, we use the winner-take-all competitive learning rule to generate a one dimensional topological map using a two layers neural network. Here, the two layers neural network structure includes one input layer and one output layer. The input layer is used to receive the six dimension feature vector which consists of three pieces of synthetic information and three pieces of labeling information. Figure 9 shows the process of one-dimensional topological map generation.

Then, we individually collected 36,000 pixel vectors with the labeled information of ships, clouds, and the sea surface, and used these collected pixel feature vectors to generate a one-dimensional topological map that follows the special loss function in Equation (13).

(13) $$f(x)=\arg\underset{j}{\min}\left\|x-w_{j}\right\|.$$

where *j* is the index of the initialization neurons which are ships, clouds and the sea surface. *x* is the input pixel feature vector with the labeled information. *f*(*x*) is the winner neuron which has the minimum Euclidean distance between the inputs and three competitive neurons. When we obtain the winner neuron, its weight is updated by Equation (14).

(14) $$w_{j}(t+1)=w_{j}(t)+\eta (t)h_{j,f(x\left(t\right))}(x(t)-w_{j}(t)).$$

Here, *t* is the iteration number of the whole training process, and *η*(*t*) is the learning rate, which changes iteratively. *h_j,f_*_(*x*(*t*))_ is the topological neighborhood function for the weight updates. The learning rate and topological neighborhood function in Equation (14) can be defined by Equations (15) and (16), respectively.

(15) $$\eta (t)=\eta_{0}\exp(-\frac{t}{\tau_{1}}).$$

(16) $$h_{j,f(x(t))}=\exp(-\frac{1}{2\cdot {\left[\sigma_{0}\exp(-\frac{t}{\tau_{2}})\right]}^{2}}).$$

In Equations (15) and (16), we used k-fold cross-validation method [37,38] to ensure and verify learning parameters. Here, “k” is set to 10, which is “10-fold cross-validation”. First step, we initialized $\sigma_{0}$, $\tau_{1}$, and $\tau_{2}$ as a small number range from 0 to 1. Then, the 36,000 pixel level feature vectors are separated as 10 sub-datasets. Nine of sub-datasets are used to train SFCM (i.e., automatically getting clustering center and controlling distance by one dimensional topologic map), and remaining sub-dataset is used to test trained SFCM performance. If the performance is not good, $\sigma_{0}$ would be adjusted. The second step involves changing the testing dataset for another sub-dataset and repeating the first step 10 times. Here, $\sigma_{0}$ is a very important parameter which has to be ensured to impact SFCM final performance. Other parameters such as $\tau_{1}$ and $\tau_{2}$ only affect the speed of convergence. *t* is the iteration number. In Equation (15), we set $\tau_{1}$ as 0.4. *η*_0_ is the initial learning rate, which is set as 0.12. In Equation (16), $\sigma_{0}$ is the initial variance of the Gaussian function, which set as 0.3. $\tau_{2}$ is set to 0.6. Through the processes that were mentioned before, we can automatically obtain the one-dimensional topological map. Next, we chose the updated competitive neuron of ships without labeled information as the clustering center coordinates in Equation (12). Then, we chose the minimum distance between the clouds or the sea surface compared with the ship’s neuron vector in the one dimension topological map as the controlling distance *d* in Equation (12). Finally, we set up a SFCM in Equation (12) that can analyze and extract ship candidates pixel by pixel from the defined complex local scene.

### 2.4. HOG and SVM Classifier for Ship Candidate Confirmation

After RSM and SFCM ship candidate extraction, the simple features—the area and the ratio of the length to the width—were used to eliminate false alarms [8,11,17]. Next, the Radon transform was used to rotate the remaining candidates to their major direction. Finally, the 1200 ship and 1200 false alarms HOG feature-trained SVM classifier was used to achieve the ship candidate confirmation [11].

## 3. Experiments and Results Discussion

In this section, we apply 255 SPOT-5 images with 5-m spatial resolution 4069 × 4096 sizes and 210 GF-1 images with 2-m spatial resolution and 8192 × 8192 sizes to test the performance of the proposed ship detection framework. Then, the collected SPOT5 and GF-1 data are formatted into regular size of 4096 × 4096, and the scenes display the possible variable situations (i.e., big cloud covering, broken clouds, strong waves, quiet seas, and fleets of ships). The computation environment uses the Windows 7 operating system with a 2.00 GHz Intel^®^ Corel^TM^ i7-4500U CPU and 7.71 GB RAM. Then, all of the ship detection methods are tested using MATLAB 2016a. Finally, we choose 1341 ship samples and 10,000 false alarms to test this paper’s proposed method and the state of the art ship detection methods. The evaluation indexes are defined as Equations (17) and (18).

(17) $$Recall=\frac{N_{DT}}{N_{TS}}.$$

(18) $$Precision=\frac{N_{DT}}{\left(N_{DT}+N_{DF}\right)}.$$

In Equations (17) and (18), *N_DT_* is the positive samples that are predicted as positive. *N_TS_* is the total number of predicted positive samples. *N_DF_* is the negative samples that are predicted as positive. Next, we use *Recall* and *Precision* to evaluate our proposed method and the other compared methods. The first section conducts the locally oriented scene characteristics analysis to find its optimal parameters. The second section discusses the RSM candidate extraction performance. The third section discusses the SFCM candidate extraction performance. The last section analyzes and compares this paper’s proposed ocean ship detection framework with the existing methods.

### 3.1. Real-Time Processing Factor Discussion of Locally Oriented Scenes Character Analysis

In this section, the real-time processing factor of the fast ocean scene partitioning method will be discussed. Since the regular input image size is fixed as 4096 × 4096, we have to find a suitable local scene size to define and analyze the complexity of local scenes using our proposed analysis method. Here, the smaller local scene size setting can generate more local scenes, which would affect the timeliness of real-time processing. If the local scene size is larger, most of the complex local scenes would be defined as simple local scenes. This would result in a bad detection performance since the RSM candidate extraction is not suitable for complex local scenes, which is discussed in detailed in Section 3.2. Table 1 shows the performance of the fast ocean scene partitioning method using different local scene sizes and the cells analysis method.

Here, the different sizes of simple local scenes are collected as positive samples, and the different size of complex local scenes are collected as negative samples. From Table 1, as the local scene size increases, the *Recall* and *Precision* ratios decrease. Therefore, a suitable local scene size setting is important for input images processing timeliness and scene partitioning accuracy. When the local scene size is smaller, it would spend much more time to achieve the whole scene partitioning, and when the local scene size is larger, most complex local scenes are wrongly defined as simple local scenes, which reduces the accuracy of the proposed method of defining local scenes. Then, in Table 1, we also find that the size of cells from 3 × 3 to 7 × 7 have less impact on defining local scenes. Therefore, we chose the 162 × 162 local scene size and the 3 × 3 cellular structure to analyze the local scene characteristics since *R* in (8) is a factor to balance the timeliness and accuracy requirements of this paper’s proposed ocean ship detection framework. Here, we also have to choose a suitable value of *R* to enable the proposed ship detection framework to meet real-time processing requirements. Table 2 shows the optimal *R* factor for the 162 × 162 local scene and the 3 × 3 cellular analysis structure.

From Table 2, we can see that when *R* is equal to 5, most of the simple local scenes are defined as complex local scenes, and when *R* is equal to 40, most of the complex local scenes are defined as simple local scenes. If most of the complex local scenes are defined as simple local scenes, the RSM would result in a bad detection performance, which would increase the time consumption of the ship pixel feature clustering analysis. Therefore, we have to choose a suitable threshold *R* to balance the timeliness and detection accuracy. In Table 2, we can find that when *R* is equal to 15, most of the simple local scenes are defined correctly, and the proposed locally oriented scene characteristics analysis would ensure high detection accuracy and that real-time processing requirements were met.

### 3.2. Performance Discussion of RSM

**Figure 10:**
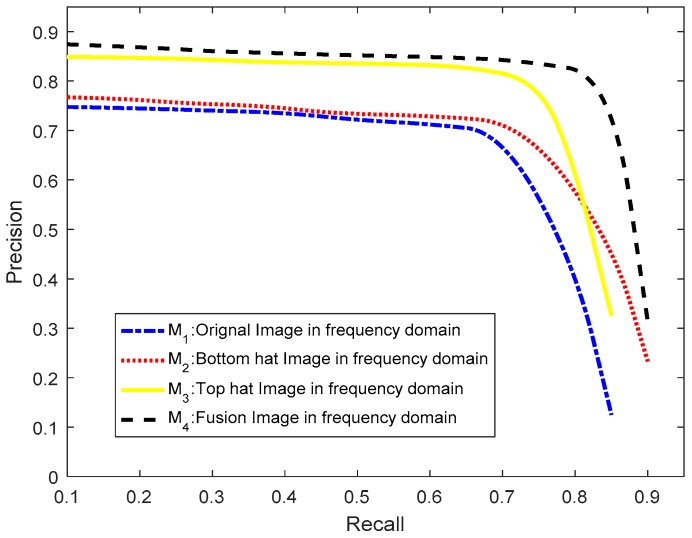
Fusion image effect for ship candidate extraction performance analysis.

In this section, we use 3215 simple and complex local scenes that are 162 × 162 in size to evaluate the RSM’s performance. First, we have to evaluate the fusion image effect for the frequency domain pulse response analysis. Figure 10 shows effect of the fusion image.

In Figure 10, M_1_ is the blue curve, which is the original image testing analysis in the frequency domain. Ocean ships with low contrast ratio cannot have pulse responses in the frequency domain. Therefore, if the original image is analyzed in the frequency domain, the low contrast ratio ship targets would be lost during the candidate extraction stage, which would lead to a bad ship detection performance. M_2_ (red curve), which is the bottom hat transform image analysis in the frequency domain, can enhance darker ships. M_3_ (yellow curve), which is the top hat transform image analysis in the frequency domain, has a better promotional effect than M_2_ because ocean ships often appear brighter in gray level remote sensing images and the top hat transform can enhance the brighter ship targets as pulse responses in the frequency domain. M_4_ (black curve), which is the fusion image analysis in frequency domain, can provide a better performance and adapt to brighter or darker low contrast ratios. 

We analyze 3215 simple and complex local scenes, which includes 1215 quiet sea surface simple local scenes and 2000 complex local scenes. The 2000 complex local scenes include 500 images with fleets of ships, 500 images with broken clouds, 500 images with heavy clouds, and 500 images with strong waves. In addition, these local scenes include 300 images of ships. We individually use these different kinds of local scenes to test the RSM ship candidate extraction performance shown in Table 3.

From Table 3, we see that the proposed RSM is more effective for the quiet sea surface situation and that there is a bad ship candidate extraction performance for complex local scenes. However, for arbitrary kinds of local scenes, the proposed method is good for real-time processing. Nevertheless, for bright broken clouds, strong ocean waves and heavy clouds, the proposed RSM would result in lost ship candidates. Therefore, since the proposed RSM is an efficient candidate extraction method for simple local scenes, the RSM should be employed for consistent real-time detection of ocean ships. Then, the complex local scenes would be processed by the SFCM.

### 3.3. Performance Discussion of SFCM

**Figure 11:**
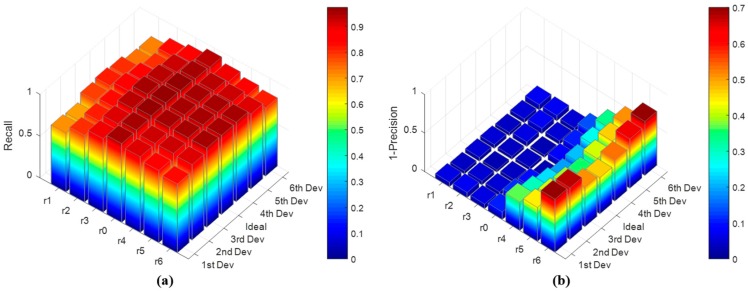
Clustering center coordinate and controlling distance of the ship feature clustering model (SFCM). (**a**) is the Recall rates of the SFCM detection performance with different clustering centers and controlling distances and (**b**) is the 1-Precision rates of the SFCM detection performance with different clustering centers and controlling distances.

For the optimal parameter performance of the SFCM. The clustering center coordinates and the controlling distance can be automatically obtained from the defined two layer neural network structure. Then, we discuss how changes in the clustering center coordinates and the controlling distance impact the detection performance in complex local scenes using *Recall* and *Precision* indexes. For the SFCM, ship pixels are positive samples, and other nonship pixels are negative samples. Figure 11 shows the SFCM ship detection performance.

**Figure 12:**
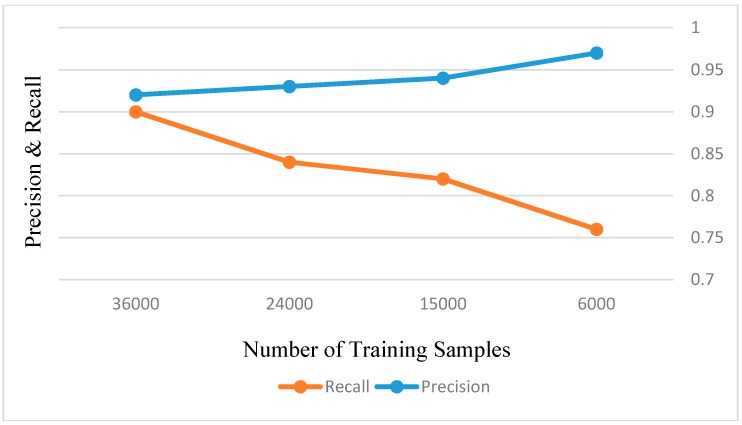
Number of training samples for SFCM training analysis.

In Figure 11, we illustrate how changes in the normal clustering center and controlling distance impact the detection performance. Here, *r*_0_ is the normal controlling distance which is obtained from the trained one-dimension topological map. *r*_3_, *r*_2_, and *r*_1_ are the controlling distances which are less than *r*_0_ and decrease by an acceptable interval. *r*_4_, *r*_5_, and *r*_6_ are the controlling distances which are more than *r*_0_ and increase by an acceptable interval. *1st Dev*, *2nd Dev*, and *3rd Dev* are the clustering center coordinates which are generated from the ideal clustering center coordinates and decrease by an acceptable interval. *4st Dev*, *5nd Dev*, and *6rd Dev* are the clustering center coordinates which are produced by the ideal clustering center coordinates and increase by an acceptable interval. Here, we chose 0.5 as a small acceptable interval value. From Figure 11, we can see the changing clustering center coordinates have less impact on the ship detection performance, and the changing controlling distance has a significant impact on the *Recall* and *Precision* rates. Therefore, we selected the ship pixel feature vector as the initialization neuron. Then, if we use a large amount of labeled pixel feature vector samples to train the one-dimension topological map, we can obtain more accurate controlling distance values to complete the accurate ship candidate extraction. However, how many samples are used to train SFCM, which is discussed in Figure 12.

From Figure 12, the 36,000 pixel feature vector training samples can improve SFCM performance more than other less samples. Therefore, we choose the 36,000 pixel feature vector as the dataset to train SFCM.

Next, we employ 3215 simple and complex local scenes of 162 × 162 in size to evaluate the SFCM’s effectiveness. Table 4 shows the performance of the SFCM applied to all types of local scenes.

From Table 4, we can see that the proposed SFCM has a good performance for ship candidate extraction in complex local scenes. Due to using a large amount of labeled pixel level training data to establish the SFCM, the ship pixels can be extracted from background interference, and the timeliness requirement is satisfied. In general, our proposed SFCM is suitable for a real-time complex local scene ship candidate extraction processing system.

### 3.4. Ocean Ship Detection Result Comparing

In this section, first we discuss this paper’s proposed ocean ship detection framework, and then, state-of-the-art ocean ship detection methods are employed as comparison to validate the efficiency of the proposed method using SPOT 5 and GF-2 ocean scene remote sensing images. The *Recall* and *Precision* are also employed as the evaluation indexes. Table 5 shows the performance of this paper’s proposed ocean ship detection framework and the other comparison methods.

**Figure 13:**
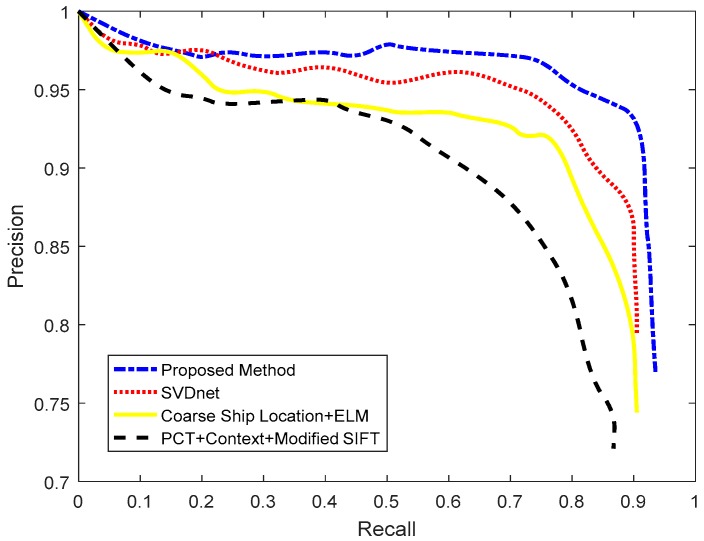
Ocean ship detection performances analysis.

Table 5 shows each stage of the proposed ocean ship detection framework and how it affects detection performance. We can see that the RSM has good timeliness, but poor ocean ship detection performance. The SFCM has better performance, but it is time consuming. Therefore, the real-time processing factor *R* is set as 15, which can balance the detection performance and timeliness requirement. Here, the HOG feature descriptor eliminates false alarms by using the trained SVM classifier which can improve the detection performance. A previous described method [9] employed the PCT for ship candidate extraction, and the PCT had a poor performance. When the ocean ship target size or gray level change and there is complex background interference, the PCT based method would lead to missed target ship. Therefore, [9] would have a lower *Recall* rate than other ocean ship detection methods. A different previously described method [13] employed the sea–land segmentation and ship locating criteria for coarse ship location detection, which would generate many false suspected candidates. Thus, this method [13] needs a powerful feature descriptor and stronger classifier to achieve ship candidate confirmation. The same method [13] employed the fusion Stacked Denoising Autoencoder (SDA) feature and the Extreme Learning Machine (ELM) to build a powerful ocean ship classifier. However, due to the wide view that the ocean scenes remote sensing images have, 2–5 m spatial resolution, and that the ocean ships do not have enough validated feature information to the support the powerful feature descriptor, this method has limited detection capability. A different previously described method [15] established a two layer Convolutional Neural Network (CNN) ocean ship detection structure based on the Singular Value Decomposition (SVD) convolutional weight initialization. It is a popular method of ocean ship detection because of its simple structure and powerful feature description. Nevertheless, this method [15] needs considerable labeled ocean ship data to train a robust model, and the manual annotation of a wide view of ocean ships is very difficult. Therefore, this method’s [15] performance is related to the amount of annotated training data. In addition, both aforementioned methods [13,15] have bad performance when an ocean ship’s gray level, scale, and/or contrast ratio change, and they often fail in more complex local ocean scene situations (i.e., broken clouds, heavy cloud cover, and strong waves). For this paper’s proposed method, we implement the real-time processing factor *R* to balance the timeliness and detection performance, which separates full scenes as simple and complex to ensure the targeted processing. Figure 13 and Table 6 show the details of the comparison and analysis of the proposed method and the state of the art methods.

**Figure 14:**
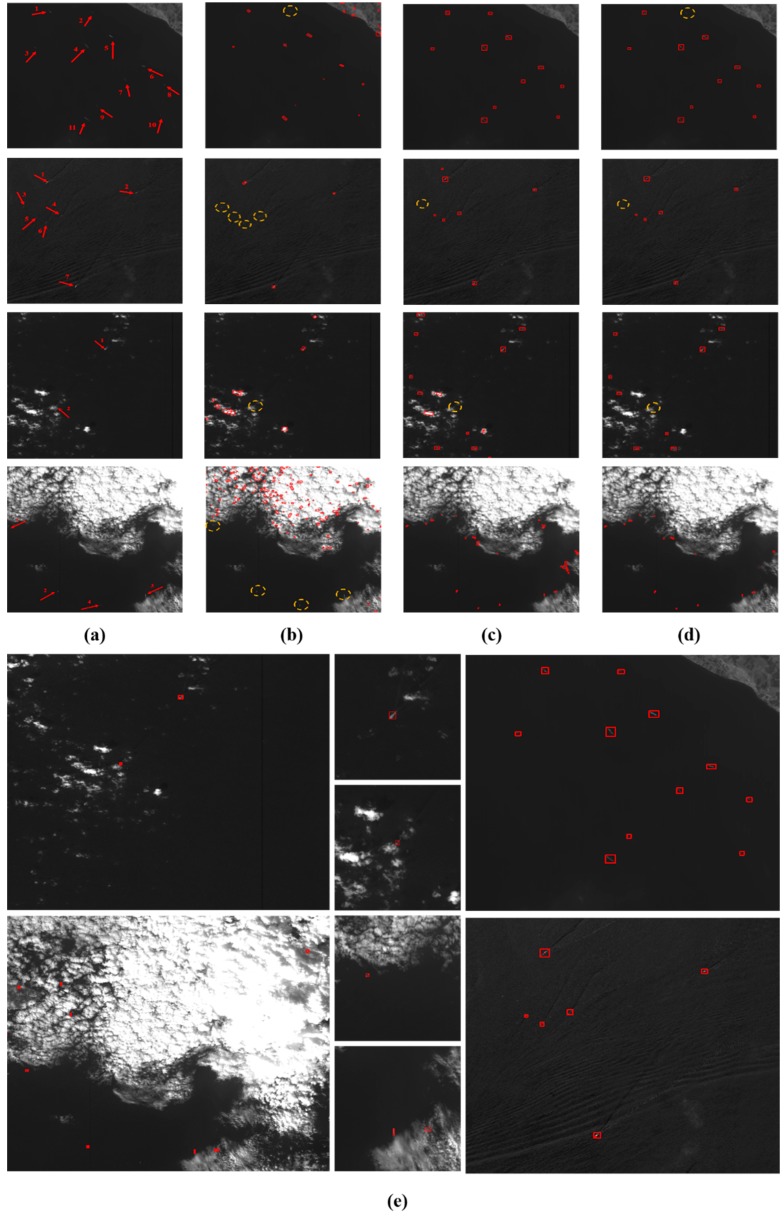
Comparison of methods of ocean ship detection performance with different scene cases. (**a**) original images; (**b**) PCT + Context + Modified modified scale invariant feature transform (SIFT) detection results; (**c**) Coarse Ship location + ELM detection results; (**d**) SVDnet detection results; and (**e**) proposed ocean ship detection framework performance with the local scene cases.

Figure 13 shows the general detection performance of the proposed method and other state-of-the-art methods. Here, the method proposed by this paper has better detection performance than the state of the art methods. From Table 6, the state of the art methods [9,13,15] have bad performance with complex local scenes. Then, the size of the ships can also affect the ship detection performance. As shown in Table 6, this paper proposed an ocean ship detection framework that can adapt to any local situations, and setting a suitable real-time processing factor *R* can ensure the detection performance and timeliness requirement. Here, we chose a series of ocean scenes to test the comparison methods. The results are shown in Figure 14. In Figure 14, the red arrow in (a) is the ground truth, which is the total number of real ships in ocean scenes. The red detection window in (b–d) shows the detection performance of the comparison methods, and the yellow dashed circle shows the missing ship targets in whole scenes. In Figure 14a, we analyze ocean scene remote sensing images including quiet seas, strong waves, broken clouds, and heavy cloud cover situations, and the ocean ship’s size and gray level variations. From (b–d), we can see that the PCT + Context + Modified SIFT [9] produce many false alarms and missing ship targets. Coarse ship location + ELM [13] and SVDnet [15] have good detection performance, but for more complex scenes, they both have false alarms and missing ship targets. Figure 14e shows this paper’s proposed ocean ship detection framework’s general performance and detailed local detection results. From Figure 14, here, the red detection window in clouds are the false alarms. We can see that our method has better performance than the state of the art methods, and the detection results meet the real-time processing precision requirement. For the proposed ocean ship detection method, we used a Xilinx XC6VLX130T to implement the proposed algorithm. The resource occupancy is approximate 34% and the average processing time for an image is approximate 1.1 s under the working clock of 80 MHz.

## 4. Conclusions

For large view ocean ship detection, the task must meet the detection timeliness and accuracy requirements based on ship movements and certain complex background interferences. In this study, the state of the art methods focus on integrating ship candidate extraction and a strong classifier with a powerful feature description in a robust ship description model. If the robust ship description model can adapt to more complex ocean scenes, it will require a more complex calculation model that will not be timely. However, the state of the art methods do not provide reliable detection results for more complex ocean scenes. Due to the poor timeliness and detection accuracy for more complex ocean scenes, the novelty of this paper is that we designed a real-time processing factor, *R*, to guide the targeted processing of simple or complex local scenes. Next, the RSM and SFCM are proposed to meet the real-time ocean ship detection requirement. We can consider the actual application requirement to adjust the real-time balance factor *R*. With respect to *R*, the complex scenes would be addressed with the pixel level analysis SFCM in order to improve the integral detection performance, and it is very suitable for a real-time ocean ship detection system. Finally, our future work will aim at building a self-adapting mechanism to separate a whole scene as several kinds of local scenes that are not related to the balance factor *R*. Then, we will improve the timeliness performance of the ocean ship detection model.

## Figures and Tables

**Table 1:** Fast local scene partitioning performance with varied local scene sizes.

Local Scene Size	Size of Cells	*Recall*	*Precision*	Times (s)/Scene
18 × 18	3 × 3	96.42%	100%	2.000
	5 × 5	95.97%	100%	2.450
	7 × 7	94.32%	100%	2.770
54 × 54	3 × 3	91.77%	100%	1.170
	5 × 5	92.56%	100%	1.370
	7 × 7	91.05%	100%	1.690
162 × 162	3 × 3	87.84%	100%	0.150
	5 × 5	86.95%	100%	0.660
	7 × 7	84.73%	100%	0.790
486 × 486	3 × 3	70.79%	100%	0.050
	5 × 5	71.44%	100%	0.057
	7 × 7	69.94%	100%	0.077
1458 × 1458	3 × 3	42.16%	100%	0.003
	5 × 5	41.95%	100%	0.009
	7 × 7	41.04%	100%	0.014

**Table 2:** Real-time processing balance factor *R* evaluation.

*R*	5	10	15	20	25	30	35	40
*Precision*	100%	100%	100%	88.17%	65.19%	53.57%	36.14%	25.26%
*Recall*	23.32%	44.72%	87.21%	100%	100%	100%	100%	100%

**Table 3:** RSM ship candidate extraction performance.

Local Scene Character	Quiet Sea Surface	Fleet of Ships	Broken Clouds	Heavy Clouds	Strong Waves
*Precision*	92.5%	84.4%	75.3%	80.4%	66.8%
*Recall*	90.0%	77.5%	64.9%	67.7%	65.7%
Time (s)/local scene	0.025	0.043	0.032	0.061	0.042

**Table 4:** SFCM ship candidate extraction performance.

Local Scene Character	Quiet Sea Surface	Fleet of Ships	Broken Clouds	Heavy Clouds	Strong Waves
*Precision*	94.3%	92.6%	89.7%	90.0%	90.8%
*Recall*	95.0%	93.5%	90.5%	91.2%	89.7%
Time (s)/local scene	0.64	0.74	0.82	0.58	0.70

**Table 5:** Proposed ocean ship detection performance analysis.

Method	*R*	*Precision*	*Recall*	Time (s)/Scene
RSM	/	79.3%	72.4%	1.25
SFCM	/	89.5%	93.0%	4.72
(RSM + SFCM)_1_	5	90.7%	91.3%	3.49
(RSM + SFCM)_2_	15	89.4%	90.2%	2.46
(RSM +S FCM + HOG)_1_	5	94.5%	93.5%	3.83
(RSM + SFCM + HOG)_2_	15	92.9%	91.7%	2.96
PCT + Context + Modified SIFT [9]	/	79.8%	78.3%	3.29
Coarse Ship Location + ELM [13]	/	87.4%	82.8%	7.48
SVDnet [15]	/	89.8%	86.1%	9.89

**Table 6:** Detailed analysis and comparison with state of the art methods.

Local Scene	Target Size	Total Number of Real Ships	Total Number of Ships Detected	*Precision*	Number of Falsely Detected Ships	*Recall*
***Proposed method***						
Quiet sea	Large	45	43	96.50%	5	93.88%
	Small	102	100			
Texture sea	Large	37	35	92.93%	7	92.00%
	Small	63	64			
Cluster sea	Large	32	35	89.47%	8	89.47%
	Small	44	41			
***PCT + Context + Modified SIFT Method*** [9]						
Quiet sea	Large	45	37	95.17%	7	93.87%
	Small	102	108			
Texture sea	Large	37	40	84.69%	15	83.00%
	Small	63	58			
Cluster sea	Large	32	30	59.46%	30	57.89%
	Small	44	44			
***Coarse Ship Location + ELM Method*** [13]						
Quiet sea	Large	45	44	97.20%	4	94.56%
	Small	102	99			
Texture sea	Large	37	35	88.42%	11	84.00%
	Small	63	60			
Cluster sea	Large	32	30	76.61%	19	69.74%
	Small	44	42			
***SVDnet Method*** [15]						
Quiet sea	Large	45	43	95.80%	6	93.20%
	Small	102	100			
Texture sea	Large	37	41	89.11%	11	90.00%
	Small	63	60			
Cluster sea	Large	32	30	84.43%	13	75.00%
	Small	44	40			
